# Invasive *Candida* pneumonia, in association with *Candida* esophagitis and gastritis, in a presumably immunocompetent patient

**DOI:** 10.4322/acr.2023.443

**Published:** 2023-08-07

**Authors:** Devon Jackson, Lamarque Coke, Kamilah Fernandez, Kathriel Brister

**Affiliations:** 1 Howard University Hospital (HUH), Department of Pathology, Washington, DC, USA; 2 Howard University College of Medicine (HUCM), Washington, DC, USA

**Keywords:** Candida, pneumonia, esophagitis, gastritis

## Abstract

*Candida* pneumonia remains a difficult diagnosis and is most common in immunocompromised individuals. It has been rarely reported in immunocompetent individuals. We present a case of unsuspected *Candida* pneumonia associated with *Candida* esophagitis and gastritis discovered on postmortem examination in a presumably immunocompetent patient. The patient was a 71-year-old male who presented with chest pain and was subsequently found to have a myocardial infarction treated with angioplasty and drug-eluting stent placement. The patient’s recovery was complicated by pneumonia refractory to antibiotics, and he went on to experience acute hypoxic respiratory failure, sepsis, disseminated intravascular coagulation (DIC), and ultimately expired. Autopsy revealed evidence of myocardial infarction as well as unsuspected *Candida albicans* pneumonia, esophagitis, and gastritis. Our case highlights how a presumably immunocompetent individual can develop this infection and how *Candida* esophagitis and *Candida* gastritis can be seen in association with *Candida* pneumonia. Due to the difficulty in diagnosing *Candida* pneumonia antemortem, autopsies provide a key opportunity to better understand these cases and the factors that may contribute to their development.

## INTRODUCTION

*Candida* pneumonia remains a difficult diagnosis, especially in the antemortem setting. *Candida* is a frequent contaminant of sputum and bronchoalveolar lavage (BAL) specimens hindering its identification in the rare cases in which it is the causative agent of serious pulmonary disease. Autopsies provide an opportunity to confirm these cases and better elucidate the incidence of this condition, as microscopic demonstration of yeast invasion in lung tissue is required. *Candida* pneumonia exists in two forms. The primary form commonly occurs following the aspiration of colonized oropharyngeal or gastric contents. The secondary form, which is more common than the primary form, occurs primarily as a result of seeding of the lungs secondary to hematogenous dissemination.^1,2^ *Candida* pneumonia is most commonly reported in severely immunocompromised individuals, extremely low birth weight infants, and patients with malignant tumors.^3^ It has been rarely reported in immunocompetent individuals. We present a case of unsuspected *Candida* pneumonia associated with *Candida* esophagitis and gastritis discovered on postmortem examination in a presumably immunocompetent patient.

## CASE REPORT

A 71-year-old man presented with chest pain for one-day duration. His medical history included hypertension (HTN), type 2 diabetes mellitus (DM), and pulmonary fibrosis (PF). He took medications for HTN, DM, and hypercholesterolemia. However, he was not currently taking any medications related to the history of PF. His social history was notable for daily cigarette smoking (1 pack every 1.5 days) and daily marijuana use (1 joint per day). On hospital day (HD) #1, he reported his chest pain as constant, non-radiating, and left-sided. He had associated nausea and coughing. An electrocardiogram (EKG) showed nonspecific ST and T wave abnormality in the anterolateral leads. Troponin level was elevated at 3.763 ng/mL (RR; 0.00-0.03 ng/mL). A computed tomogram (CT) of the chest showed hyperinflation of the lungs with bullous changes in the lung apices. There was a diffuse interstitial process in the remainder of the lungs, worse in the right middle and right lower lobes.

Acute coronary syndrome protocol was initiated with repeat EKG showing inferior infarction with acute myocardial infarction (MI)/ST elevation MI and repeat troponin of 25. The patient underwent a coronary angiogram, revealing a mid-right coronary artery with 100% occlusion. Balloon angioplasty and drug-eluting stent placement were performed. The patient started desaturating and developed leukocytosis (with initial blood cultures growing *Staphylococcus epidermidis*) as well as acute kidney injury. He was administered vancomycin, cefepime, and doxycycline. White blood cell count and creatinine level improved, but repeat CT chest imaging showed the development of moderate bilateral pleural effusions, a moderate pericardial effusion, and possible pneumonia. The patient was subsequently transferred to the intensive care unit for acute hypoxic respiratory failure.

On HD #8, his oxygen requirements improved, but he subsequently developed right-sided abdominal pain. Liver function tests revealed high levels of aminotransferases, and a follow-up CT showed mild wall thickening involving the descending colon. Consecutive courses of clindamycin and meropenem were begun. The patient developed worsening dyspnea and fluid overload, requiring intravenous furosemide and thoracentesis. An echocardiogram revealed new right ventricular heart failure and preserved left ventricle ejection fraction (LVEF > 70%). On HD #18, a right ventricular assist device (RVAD) was placed. The patient subsequently developed cough productive of dark-colored sputum. On HD #24, he became severely hypotensive with elevated lactic acid. Vasopressors were initiated. A CT showed findings suggesting bowel ischemia; however, surgical intervention was not recommended due to the patient’s increasing vasopressor requirements. He became somnolent, unresponsive and was intubated with concern for aspiration on intubation. Coffee ground material was subsequently identified in the nasogastric (NG) tube. Due to hemodynamic instability, endoscopy was not performed. The patient had worsening acidosis, coagulopathy, and thrombocytopenia. On HD #26, the patient expired after coding three times.

## AUTOPSY FINDINGS

**Figure 1:**
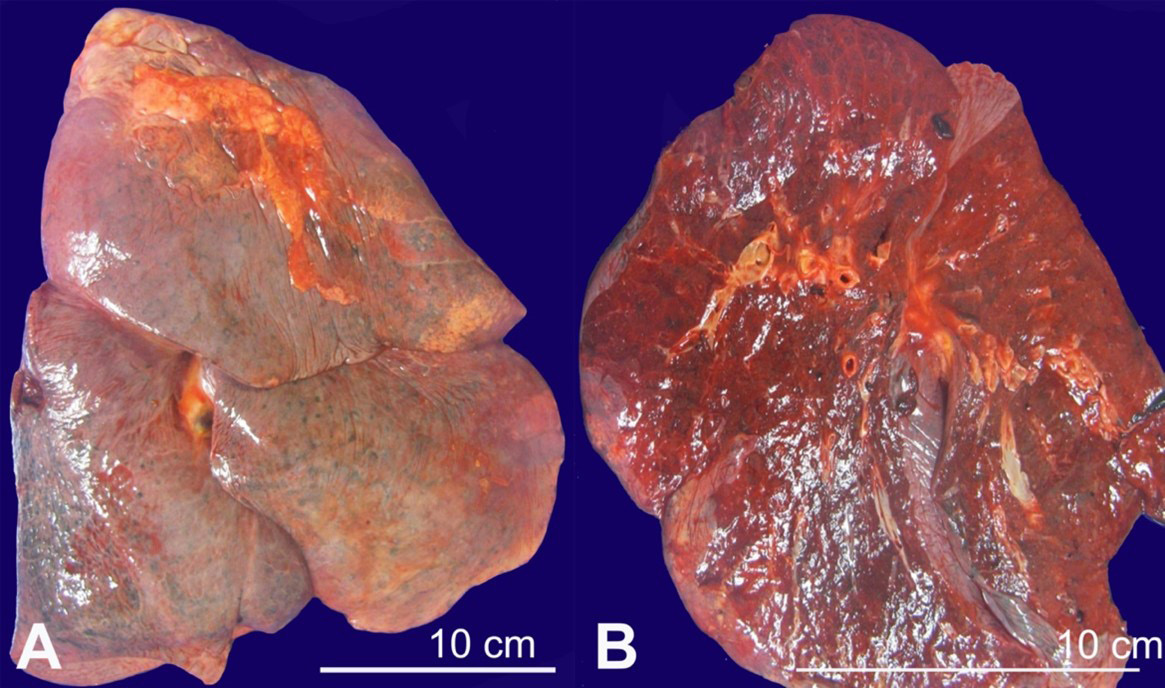
Gross images of the right lung. **A -** Anterior view of the lung with flaccid apical bullae; **B -** Cut surface of the lung showing diffuse congestion with no apparent lesions.

With the consent of the patient’s family, a complete autopsy was performed. He had a subacute myocardial infarction involving the posterior left ventricle and septum, with associated fibrinous pericarditis. There was moderate to severe atherosclerosis of the aorta and coronary arteries, with bilateral pleural and peritoneal effusions. He also had evidence of ischemic colitis. The most notable and significant findings from the autopsy were related to sepsis. He had hypercellular bone marrow for his age, liver with centrilobular necrosis and cholestasis (consistent with shock liver), and evidence of disseminated intravascular coagulopathy (microthrombi in all lobes of the right lung and in perinephric fat). An unsuspected finding was acute *Candida* pneumonia in the right lung. Grossly, a fibrous pleural adhesion was appreciated between the right lung and diaphragm. Both lungs were heavy and congested with flaccid apical bullae (Figure 1). In the right lung, the parenchyma adjacent to the bullae was notably firm on palpation.

**Figure 2:**
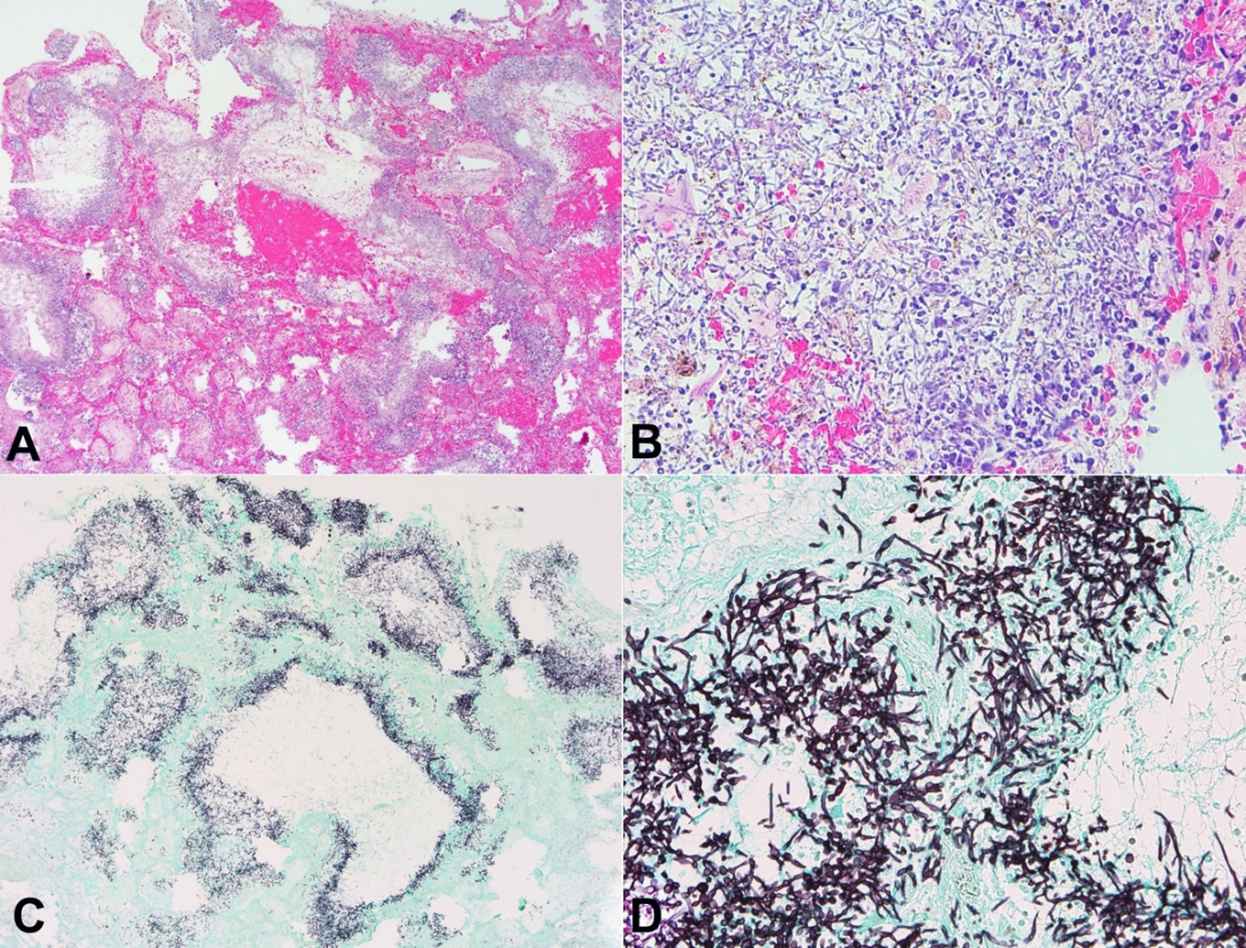
Photomicrographs of the right lung, upper lobe. **A -** Loss of normal alveolar architecture with the formation of cavitary areas, associated with inflammation, focal hyaline membrane formation, and intra-alveolar hemorrhage (40x, H&E); **B -** Abundant budding yeasts and invasive pseudohyphae are present in the cavitary areas (400x, H&E); **C** - GMS stain highlighting areas of fungal infiltration throughout the tissue (40x); **D** - Pseudohyphae and budding yeasts, morphologically consistent with *Candida*, are nicely highlighted (400x, GMS).

Microscopically, sections from the right upper and middle lobes showed acute pneumonia with cavitary lesions in association with budding yeast and invasive pseudohyphae morphologically consistent with *Candida* species. In addition to multiple cavitary lesions, the fungal forms were also associated with intra-alveolar hemorrhage and focal hyaline membrane formation (Figure 2A and 2B). A Grocott methenamine silver (GMS) stain highlighted numerous invasive pseudohyphae within the walls of the cavitary lesions (Figures 2C and 2D).

**Figure 3:**
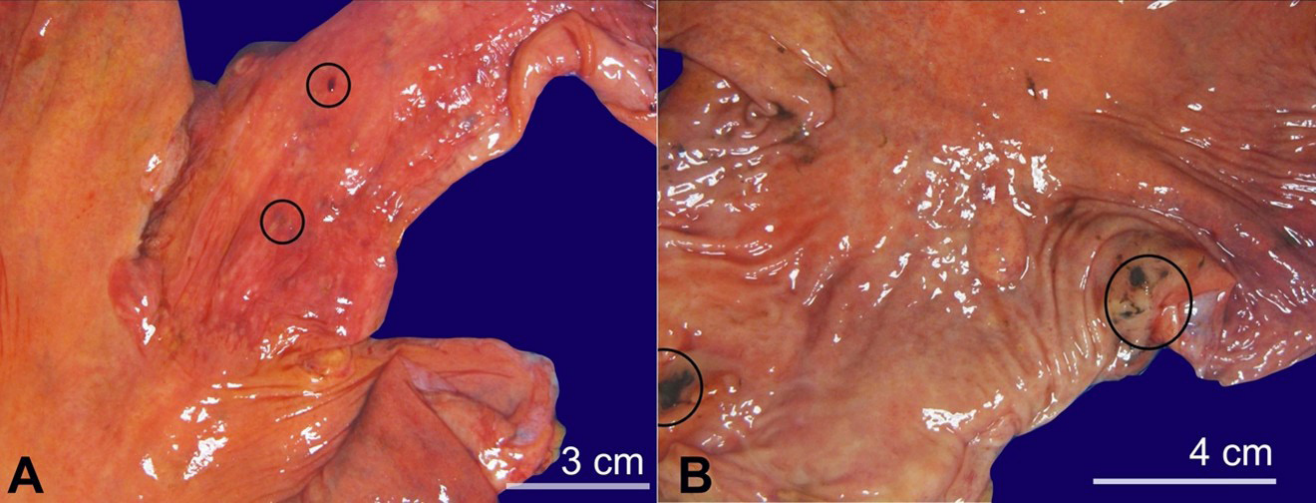
Gross images of the esophagus and stomach. **A -** Distal esophagus with focal areas of hemorrhage and ulceration; **B -** Stomach with focal areas of hyperemia and erosion.

Our routine postmortem cultures revealed the right lung tissue to be positive for *Candida* species. To determine the exact species, the respective formalin fixed paraffin-embedded (FFPE) tissue was sent to the University of Washington, where polymerase chain reaction (PCR) analysis was performed and detected *Candida albicans*. Representative sections from the esophagus and stomach also revealed fungal forms consistent with *Candida* species. Grossly, the esophagus showed focal areas with hemorrhage and ulceration (Figure 3A), and the stomach showed patchy, focal hyperemic areas (Figure 3B).

**Figure 4:**
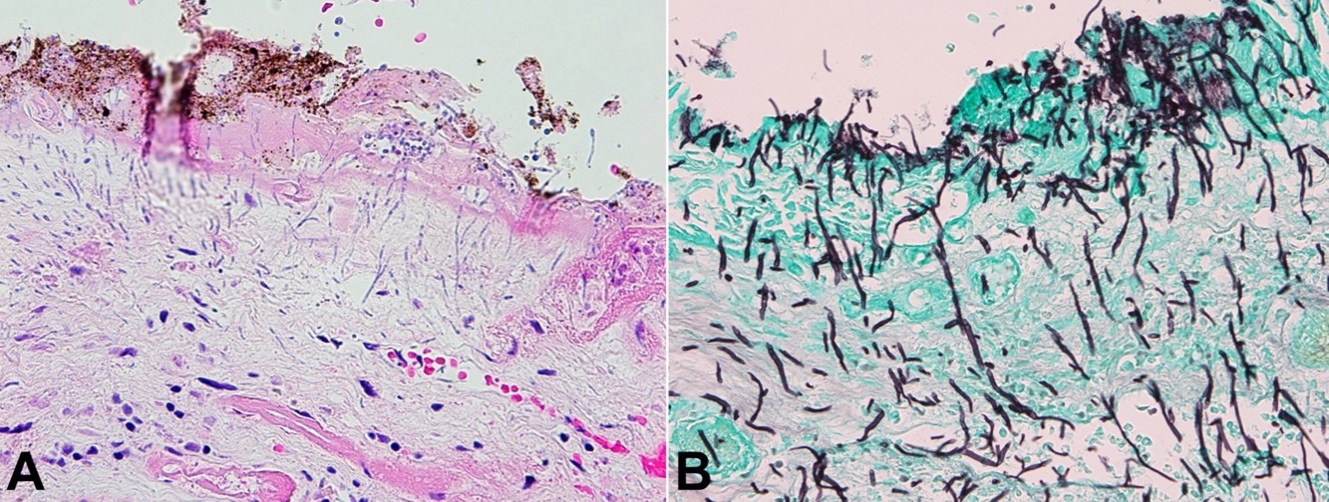
Photomicrographs of the esophagus. **A -** Area of ulceration, with fibrin and hemosiderin, showing budding yeasts and pseudohyphae infiltrating into the tissue (H&E, 400x); **B -** GMS stain highlights fungal forms with budding yeast and pseudohyphae invading the mucosa in the area of ulceration (400x).

**Figure 5:**
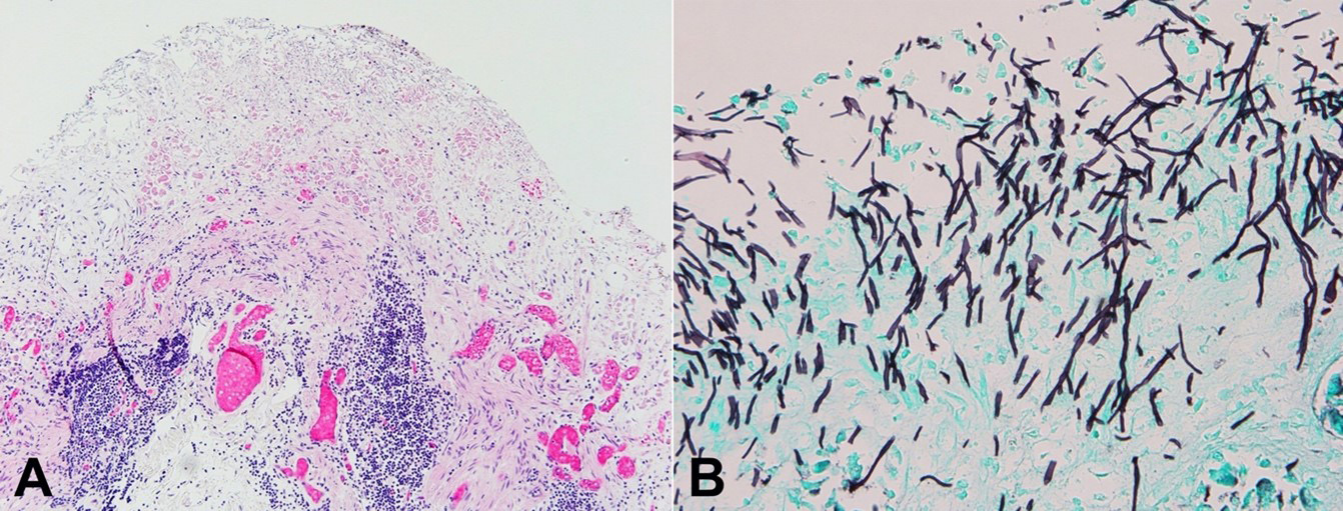
Photomicrographs of the stomach. **A -** Gastric mucosa showing areas of chronic inflammation and autolytic changes. Despite autolysis, pseudohyphae and budding yeasts, suggestive of *Candida,* can be seen within the tissue (H&E, 100x); **B -** GMS stain highlights these fungal forms invading the tissue (400x).

Representative sections from these areas showed ulceration and erosion with budding yeast and pseudohyphae morphologically consistent with *Candida* species invading the mucosa, with fungal forms highlighted on GMS stain (Figures 4A, 4B, 5A, and 5B).

The background gastric mucosa showed chronic inactive gastritis and autolytic changes. Given the destructive process of the *Candida* infection in the right lung and the additional sepsis findings found during the autopsy, the immediate cause of death for this patient was *Candida* pneumonia and sepsis.

## DISCUSSION

The most common organisms that cause fungal pneumonia are *Aspergillus*, *Cryptococcus*, *Mucor*, and *Pneumocystis jirovecii*. *Candida* encompasses more than two-thirds of cases of fungal infections, but invasive candidiasis rarely presents as *Candida* pneumonia.^4^ *Candida* pneumonia remains a rare diagnosis due to the difficulty in obtaining definitive evidence of a true infection. When Gram stains reveal yeast forms, or *Candida* is grown in sputum cultures, these are generally viewed as contaminants from colonization by these organisms.^5^ Bronchoalveolar lavages (BALs) are widely used by clinicians to confirm pneumonia and the nature of the associated pathogen. There is a defined culture threshold for bacterial colony-forming units, but this does not exist for fungal organisms. Without this clear definition, one cannot easily distinguish fungal infections from colonization.^6^ It has been proposed that cytological evidence of phagocytosis of fungal spores and special stains (e.g., GMS) can reveal invasive fungal infection on BAL, especially in patients with pneumonia responding poorly to broad-spectrum antibiotics.^7^ Common radiologic findings in cases of *Candida* pneumonia include bronchopneumonia, nodular opacities, lung abscess, and cavitary lesions. However, these findings are nonspecific. A definitive and final diagnosis requires histopathological evidence of invasive disease.

The most common histopathologic finding in *Candida* pneumonia cases is bronchopneumonia. Other findings include intra-alveolar hemorrhage and exudate, aspirated material, evidence of an inflammatory reaction with micro-abscesses (containing polymorphonuclear cells, lymphocytic cells, or both), and destruction of lung parenchyma adjacent to the abscesses.^8^ *Candida* pneumonia can occur by two routes, leading to secondary *Candida* pneumonia or primary *Candida* pneumonia. The secondary form is more common than the primary form and occurs primarily as a result of hematogenous spread to the lungs in candidiasis. Spread may come from any distant site, such as the skin, gastrointestinal tract, or extensive mucositis. Vascular invasion involving the small pulmonary arteries, arterioles, and capillaries is usually found.^6^ In the secondary form, miliary nodules are usually present histologically with a necrotizing inflammatory center and a hemorrhagic rim reflecting an intravascular distribution of fungi.^9^ In contrast, primary *Candida* pneumonia typically occurs following esophagopharyngeal or gastric aspiration. Concomitant *Candida* esophagitis and upper respiratory tract colonization are frequently found in primary cases. Given the aspiration of contents directly into the lung, the *Candida* organisms spread in the airspaces with no vascular invasion.^6^ In this form, the histologic examination may show the organisms in airways associated with an alveolar filling pattern of bronchopneumonia or, much less commonly, a bronchocentric granulomatosis or suppurative granuloma pattern.^3,9^

Colonization of the respiratory epithelium by *Candida* is considered a prerequisite for invasive infections.^4^ Risk factors for increased *Candida* load include nicotine and alcohol abuse, DM, aspiration of gastric fluid, and esophageal diverticula.^6^ Patients also appear at risk if they receive high dose steroids for nonmalignant conditions or prolonged courses of broad-spectrum antibiotics for a complicated medical illness.^10^ Even with these scenarios, *Candida* pneumonia still occurs most commonly in neutropenic patients, organ transplant recipients, and other immunocompromised hosts. At baseline, the patient, in this case, had no evidence of being immunocompromised. His history of diabetes and smoking increased his risk for *Candida* colonization, but it would not be expected to make him susceptible to invasive *Candida* infection. He had a documented history of pulmonary fibrosis, however there was no evidence he was taking steroids or other immunosuppressive drugs for this condition. We also did not find significant histopathologic evidence of pulmonary fibrosis, even after taking multiple additional lung sections. This patient had a complicated hospital course which may have contributed to disruption in his immunologic response. He was on antibiotics for sixteen consecutive days for concerns of pneumonia and colitis. He also had a poor cardiac function and underwent an invasive procedure with RVAD placement.

Toward the end of the patient’s hospital course, he notably had dark-colored sputum and coffee-ground material present in his NG tube following its placement. The dark-colored sputum may have been due to the invasive *Candida* infection in the lung. The coffee ground material can be attributed to the focal ulceration in the esophagus and the hyperemic erosive areas in the stomach found at autopsy. Ulceration and erosion of the esophageal and gastric mucosa likely disrupted the normal environment and allowed *Candida* to invade the tissue. Given the invasive forms seen histologically, these fungal organisms could have entered the blood and disseminated to the lung. However, there was no evidence of *Candida* in the pulmonary vasculature and no evidence of *Candida* in any “sterile” organs. Also, postmortem blood culture was negative for *Candida*. Disseminated candidiasis remains a clinical diagnosis, and unfortunately, blood culture is insensitive. However, postmortem blood cultures have been shown to be highly specific indicators of disseminated disease.^11^

With these findings, primary *Candida* pneumonia is favored over secondary. Outside of the lung, the esophagus and stomach were the only two areas with invasive *Candida* infection. We hypothesize that *Candida* entered this patient’s lung through aspiration. Concern for aspiration was only documented at the time of intubation, but there could have been other unwitnessed events. Considering the presence of *Candida* in the patient’s esophagus and stomach, aspiration of gastric contents is a plausible source of these fungal organisms in the lung. Evidence of *Candida* infection was only identified in the right lung. Aspirated gastric contents are more likely to end up in the right lung due to the shorter and straighter anatomy of the right mainstem bronchus. Whether primary or secondary, the microscopic and microbiology culture findings in this case support a diagnosis of *Candida* pneumonia. Our hospital microbiology lab only resulted the culture with the genus and not the species. To determine the species, we sent the FFPE tissue to an outside lab for PCR resulting in the identification of *Candida albicans*. We are aware of only two other cases in which the diagnosis of *Candida* pneumonia was confirmed by PCR, biopsy, and microbiology cultures. One case identified *Candida dubliniensis*,^12^ while the other identified *C. albicans*.^3^

*Candida albicans* is the most common pathogen in systemic and pulmonary candidiasis. The development of infections occurs when there is dysbiosis of the normal flora, immune dysfunction, and damage to the muco-intestinal barrier. One of four events is considered necessary for disseminated candidiasis - spread from fungal biofilms, direct damage of mucosal barriers, indirect translocation of the fungal cells phagocytosed by host immune cells, or direct invasion of epithelial cells into blood capillaries and vessels.^13^ *C. albicans* is present in the form of yeasts in the human microbiome, but its hyphal form is pathogenic. The hyphal form is invasive, actively penetrating host tissue and inducing endocytosis. The main origin of *C. albicans* is located in the gastrointestinal (GI) tract. Infection with *C. albicans* mostly occurs after infection of the GI system. Esophagitis caused by *C. albicans* is commonly seen and is most often superficial. However, complications and invasion with hematogenous dissemination can occur. Rarely, infections in the esophagus can be transmural and lead to perforation.^14^

*Candida* esophagitis has often been documented in association with cases of *Candida* pneumonia.^8^ However, to our knowledge, this is the first case that also has associated *Candida* gastritis. *Candida* is part of the normal flora in the stomach, but infection in the stomach is rare, usually in association with malignancy. Only a few cases have been reported. In one case report, *Candida* infection in the stomach was seen in conjunction with infection in the esophagus and duodenum. The patient had a history of chronic lymphocytic leukemia and presented with multiple mass-like ulcerations concerning malignancy, but histology revealed ulcerated mucosa, abundant granulation tissue, numerous yeasts, and pseudohyphal fungal forms consistent with *Candida*.^15^ Another case involved a large gastric ulcer identified in an immunocompetent patient who developed fever. An initial biopsy revealed *Helicobacter pylori*-associated gastritis that was treated with a combination of antibiotics. The patient’s fever did not respond, so a biopsy was repeated and demonstrated granulation tissue, numerous yeasts, and pseudohyphal fungal forms consistent with *Candida*.^16^ Why the esophagus is more prone to *Candida* infection than the stomach is still unclear, but it may be due to the protective effects of gastric acid in the stomach.^15^

As with most fungal infections, involvement of the stomach is more commonly seen in patients with underlying malignancy or those in an immunosuppressed state. It is infrequent in immunocompetent individuals. It also has not been reported in combination with *Candida* pneumonia. Definitive and probable cases of *Candida* pneumonia have been associated with aspiration, particularly in immunocompetent patients.^6,8,17^ *Candida* pneumonia is well documented in cancer and transplant patients, with hematogenous dissemination commonly seen.^8,18,19^ Invasive infection limited to the lungs or primarily involving the lungs has been rarely reported. With aspiration of oropharyngeal, esophageal, and gastric contents cited in numerous cases, aspiration of *Candida* infection from the esophagus and stomach was the most likely source of our patient’s infection.

This patient had a complicated clinical course, and fungal infection did not appear to be a clinical consideration. Initial blood cultures grew *S. epidermidis*. Blood cultures were performed three additional times and showed no growth. The patient’s respiratory status improved at one point but began worsening again. Given his poor cardiac function, his decline in respiratory function could have been attributed to heart failure and not persistent pneumonia. Imaging did not suggest worsening of his pulmonary infiltrates, but it also did not show improvement. When the patient developed coffee ground emesis, the gastroenterology team considered endoscopy but deferred it due to the patient being hemodynamically unstable. If an endoscopy had been able to be performed, biopsies of the esophagus and stomach may have yielded diagnostic tissue, and the patient could have been started on antifungal treatment. Five days prior to the coffee ground material, the patient had cough productive of dark-colored sputum. Testing of the sputum may have yielded findings of *Candida*, though it may have been dismissed as a contaminant.

In patients with concern for pneumonia or poor respiratory function with no improvement clinically or radiologically, a fungal infection should be considered as a possibility. Individuals that are presumably immunocompetent may have disruptions in their immune defenses, as we saw in this case, allowing for invasive *Candida* infection in the esophagus, stomach, and lungs. The gold standard for diagnosing *Candida* pneumonia continues to be histologic evidence of invasive disease. Many patients are not stable enough to undergo a lung biopsy, so they may never have a definitive antemortem diagnosis. Autopsies provide a key opportunity to better understand these cases and the factors that may contribute to their development. In decedents with a concerning history, such as pneumonia not responding to antibiotics, an autopsy should be recommended. With the extensive sampling of lung tissue and other organs that can be done in autopsies, more cases of *Candida* pneumonia can be identified.

## CONCLUSION

Overall, *Candida* pneumonia is a rare disorder, occurring more often in immunocompromised and immunosuppressed individuals. Immunocompetent patients can develop *Candida* pneumonia, though antemortem diagnosis is extremely challenging. Aspiration appears to be a major risk factor, but the factors contributing to this condition in immunocompetent individuals must be assessed further. We describe the complicated clinical course and pathologic findings in a case of *Candida* pneumonia, where the diagnosis was confirmed by three different modalities (PCR, microbiologic culture, and histopathology). The infection was severe and contributed to the patient’s death. While our case likely involved multiple factors contributing to the development of *Candida* pneumonia, it highlights how a presumably immunocompetent individual can develop this infection and how not only *Candida* esophagitis can be seen in relation to the disease but also *Candida* gastritis. While rare, involvement of the stomach by *Candida* should be considered in these cases.
